# Integrating Genomic and Morphological Approaches in Fish Pathology Research: The Case of Turbot (*Scophthalmus maximus*) Enteromyxosis

**DOI:** 10.3389/fgene.2019.00026

**Published:** 2019-01-31

**Authors:** Paolo Ronza, Diego Robledo, Roberto Bermúdez, Ana Paula Losada, Belén G. Pardo, Paulino Martínez, María Isabel Quiroga

**Affiliations:** ^1^Departamento de Anatomía, Producción Animal y Ciencias Clínicas Veterinarias, Universidade de Santiago de Compostela, Lugo, Spain; ^2^Royal (Dick) School of Veterinary Studies, The Roslin Institute, The University of Edinburgh, Midlothian, United Kingdom; ^3^Departamento de Zoología, Genética y Antropología Física, Universidade de Santiago de Compostela, Lugo, Spain

**Keywords:** *Scophthalmus maximus*, Myxozoa, pathogenesis, histopathology, transcriptomics

## Abstract

Enteromyxosis, caused by *Enteromyxum scophthalmi*, is one of the most devastating diseases stemming from myxozoan parasites in turbot (*Scophthalmus maximus* L.), being a limiting factor for its production. The disease develops as a cachectic syndrome, associated to catarrhal enteritis and leukocytic depletion, with morbidity and mortality rates usually reaching 100%. To date, no effective treatment exists and there are different unknown issues concerning its pathogenesis. The gross and microscopic lesions associated to enteromyxosis have been thoroughly described, and several morphopathological studies have been carried out to elucidate the mechanisms of this host-parasite interaction. More recently, efforts have been focused on a multidisciplinary approach, combining histopathology and transcriptome analysis, which has provided significant advances in the understanding of the pathogenesis of this parasitosis. RNA-Seq technology was applied at early and advanced stages of the disease on fishes histologically evaluated and classified based on their lesional degree. In the same way, the transcriptomic data were analyzed in relation to the morphopathological picture and the course of the disease. In this paper, a comprehensive review of turbot enteromyxosis is presented, starting from the disease description up to the most novel information extracted by an integrated approach on the infection mechanisms and host response. Further, we discuss ongoing strategies toward a full understanding of host-pathogen interaction and the identification of suitable biomarkers for early diagnosis and disease management strategies.

## Introduction

Turbot (*Scophthalmus maximus* L.) is a flatfish species naturally distributed throughout the European coast, from the Baltic and the Atlantic Ocean up to the Black Sea, being scarce in the Mediterranean Sea (Prado et al., 2018). Fish are important for human diet, being a good source of high-quality proteins, vitamins, and other essential nutrients, including n-3 polyunsaturated fatty acids (PUFAs) and trace minerals. Flatfish are a group of great commercial value, considered as low-fat fish (2–4% fat) with a firm, white, mild tasting flesh, highly accepted by the consumers (Cerdá and Manchado, 2013; Dong et al., 2018). The reduction of captures caused by fisheries’ exhaustion has promoted flatfish aquaculture mainly in Europe and Asia, with turbot and Japanese flounder *Paralichthys olivaceus* as the dominant species (Food and Agriculture Organization [FAO], 2016). It is a fast-growing industry, where the high appreciation by the market allows higher prices, which compensate the greater production costs of flatfish due to their land-based aquaculture systems (Cerdá and Manchado, 2013; Robledo et al., 2017a). Turbot, in particular, is a great value species that is much favored in many market segments such as white tablecloth restaurants (Bjørndal and Øiestad, 2010). The aquaculture production of this species started in the late 1970s and has experienced an important increase in the last decade. In the European Union (EU) more than 10,000 tons of turbot were produced in 2016, mostly in Spain (>70% of EU production), and in particular in Galicia (NW Spain, 99% of Spanish production; Apromar Asociación Empresarial de Acuicultura de España, 2017). Worldwide aquaculture production of turbot rose above 65,000 tons in 2015, mostly due to its quick expansion in PR China (Martínez et al., 2016) where the species was introduced in the 1990s (Lei and Liu, 2010). As for most aquaculture species, and despite being mainly produced in land-based facilities, pathogens represent the most important threat to the sustainability of turbot aquaculture. Although there has been significant progress with the development of some effective treatments and vaccines or the identification of major genomic regions associated with pathogen resistance (Martínez et al., 2016), diseases represent the main challenge that turbot farming will face in the near future.

Bacterial diseases, such as tenacibaculosis by *Tenacibaculum maritimum*, vibriosis by *Vibrio anguillarum*, edwarsiellosis by *Edwardsiella tarda*, and aeromoniasis (furunculosis) by *Aeromonas salmonicida* subsp. *salmonicida*, are among the most common causes of economic losses in aquaculture industry. Vaccination is routinely used for tenacibaculosis and vibriosis in turbot, although sometimes the complementary use of antibiotics is necessary (Avendaño-Herrera et al., 2006). In contrast, in the case of aeromoniasis and edwarsiellosis the development of successful vaccines is still under investigation (Castro et al., 2008; Coscelli et al., 2015) and several outbreaks have recently been reported in turbot farms (Lillehaug et al., 2003; Padrós et al., 2006; Qin et al., 2014). On the other hand, currently there is not a straightforward solution to tackle parasitic diseases, especially those produced by endoparasites, and they represent one of the most important threats for turbot industry. *Philasterides dicentrarchi*, the causative agent of scuticociliatosis, has been involved in severe mortality episodes in farmed turbot (Iglesias et al., 2001) and although some encouraging results have been achieved with experimental vaccines (Sanmartín et al., 2008; Palenzuela et al., 2009b), the high variability among parasite strains and the changes in the antigen surface along the infection have precluded a general protection. Current efforts are focused on obtaining more resistant or tolerant broodstock, and for that the genomic architecture of resistance to this parasite is being evaluated in the framework of the Fishboost EU project (FP7/2007–2013, ref. 613611).

Similarly, enteromyxosis, caused by the myxozoan parasite *Enteromyxum scophthalmi*, represents a major challenge to turbot production, with morbidity and mortality rates usually approaching 100% (Branson et al., 1999; Redondo et al., 2004; Quiroga et al., 2006). Despite efforts to find an effective treatment against this disease, mainly testing coccidiostatic drugs alone or in combination with antibiotics (Bermúdez et al., 2006a; Palenzuela et al., 2009a), there are still no available therapeutic measures. Current control measures are basically preventive, focused on improving husbandry strategies. A good treatment (ozone, UV and filtration) is essential for incoming and effluent water, and periodic epidemiological surveys have been suggested for early detection of the infection. Once the presence of *E. scophthalmi* is detected at farm facilities, the only available option is culling of the units where the infection was detected followed by their disinfection to minimize losses (Quiroga et al., 2006; Sitjà-Bobadilla and Palenzuela, 2012). Enteromyxosis affects turbot weighing over 50 g, with the highest prevalence observed in the range between 201 and 300 g. Mortality can be low at the beginning of the outbreak if older fish are first infected, but increases exponentially and younger fish are progressively affected. A 100% mortality is often observed in a few weeks, particularly at summer temperatures (water temperature >14°C), which have been related to a faster progress of the disease (Quiroga et al., 2006; Sitjà-Bobadilla and Palenzuela, 2012). On the other hand, turbot enteromyxosis is generally characterized by a long pre-patent period. The first clinical signs appear and the parasite is detected several weeks after the exposure (Redondo et al., 2004; Quiroga et al., 2006). Some evidences of resistance to this parasite have been reported; the origin of turbot was identified as a risk factor (Quiroga et al., 2006), and cases of fish showing protective acquired immunity after surviving the infection have been described (Sitjà-Bobadilla et al., 2004, 2007). Nonetheless, heritability for resistance to this parasite and genetic correlations with other traits have not been reported yet, and therefore, estimating these parameters for turbot enteromyxosis should be a priority to decide the best strategy for genetic breeding programs.

Transcriptome analysis is widely used as a powerful tool to gain a better understanding of the underlying pathways controlling disease progression in hosts (Sudhagar et al., 2018). A proper understanding of host-pathogen interaction is critical to devise successful disease prevention strategies, and the study of gene expression profiles is key to achieve this goal. The field of transcriptomics has constantly evolved from the first studies performed using the hybridization-based microarray technology, from full cDNA-probes to short and more precise oligo-probes. Particularly, oligo-microarrays have been employed in turbot to study the genetic response to aeromoniasis (Millán et al., 2011) and scuticociliatosis (Pardo et al., 2012). Nevertheless, the microarray technology presents some limitations, such as the requirement of a prior knowledge of gene sequences from the organism of interest, the restricted dynamical range, the low accuracy at gene families caused by cross-hybridization (particularly important in teleost due to their specific whole genome duplication), and finally the difficulties to identify alternative splice variants, essential for understanding the expression profiles of the different isoforms obtained from single genes (Wang et al., 2009).

RNA sequencing (RNA-Seq) is an evolving technology that uses next-generation sequencing (NGS) to obtain transcriptome profiles. It emerged as a rapid and effective approach for genome survey, and massive functional gene and molecular marker identification (Hrdlickova et al., 2017). In recent years, the application of RNA-Seq to biological investigations is revolutionizing the outlook and accelerating the knowledge of the eukaryotic transcriptome. In the field of pathology, RNA-Seq analysis during host–pathogen interaction allows us to deeply explore the mechanisms of infection and the defense strategies of the host, providing valuable information for developing effective targeted control and therapeutic measures. This technology has recently been employed in several investigations on fish diseases (Sudhagar et al., 2018), including vibriosis by *V. anguillarum* in turbot (Gao et al., 2016).

On the other hand, there is a paucity of works, especially in fish pathology, where transcriptomic analysis is combined with a morphopathological approach. The great amount of data generated by RNA-Seq is best exploited in an interdisciplinary approach, combining the essential expertise in bioinformatics and genetics, with a proper immunological, microbiological and pathological point of view. Particularly, tissue-based works aimed to investigate disease pathogenesis require specialists, from the experimental design, the quality assessment and characterization of the specimens to the contextual interpretation of the results in relation to the tissue and the disease under study (Berman et al., 2012).

In this sense, the assembly and annotation of the turbot genome (Figueras et al., 2016) has represented a landmark for the application of RNA-Seq technologies, facilitating the mapping and annotation of sequencing data, which translates into more accurate and comprehensive results. A new refined version of the turbot genome has recently been released and made available at the NCBI genome database (Maroso et al., 2018; GCA_003186165.1). In the case of turbot enteromyxosis, RNA-Seq analysis was applied to get insights into the pathogenesis of this threatening disease, selecting the specimens based on a histological evaluation and grading of the lesions observed after an experimental infection, and analyzing the data in an integrated framework which considered the evolution of the parasitosis as evidenced by tissue lesion (Robledo et al., 2014; Ronza et al., 2016).

In this paper a thorough review of turbot enteromyxosis is presented, discussing the recent advances in host-parasite interaction obtained by integrating the application of genomics and morphopathological techniques.

## Turbot Enteromyxosis

### Disease Description and Gross Pathology

The occurrence of an emaciative condition in farmed turbot was increasingly reported in NW Spain in the 1990s. The first studies promptly associated the disease to the presence of a myxozoan parasite in the gastrointestinal tract of the affected fish (Branson et al., 1999). The genus *Enteromyxum* was then proposed by Palenzuela et al. (2002) as a result of the study of the causal agent involved in the disease, and the parasite named as *E. scophthalmi*. The new genus was demonstrated by phylogenetic analysis using ribosomal RNA, and the two species previously known as *Myxidium leei* and *M. fugu* were included in this genus (Palenzuela et al., 2002; Yanagida et al., 2004). *E. scophthalmi, E. leei*, and *E. fugu* are still the only three known species of this genus of myxosporean parasites (Sitjà-Bobadilla and Palenzuela, 2012).

Although the presence of an intermediate invertebrate host is hypothesized for all Myxozoa, which usually present a diphasic life cycle alternating between invertebrate (actinospore phase) and vertebrate (myxospore phase) hosts (Kent et al., 2001; Lom and Dykova, 2006), this intermediate host is still to be discovered for *Enteromyxum* spp. On the other side, it has been shown that the three species present direct fish-to-fish transmission of the vegetative stages or trophozoites (Diamant, 1997; Redondo et al., 2002; Yasuda et al., 2002).

*Enteromyxum fugu* does not represent a relevant threat for its host Tiger puffer (*Takifugu rubripes*) (Tun et al., 2002; Yanagida et al., 2006), while *E. leei* and *E. scophthalmi* have a great impact in marine aquaculture. The infection is associated to a cachectic syndrome producing high mortality and deterioration of performance indicators, causing important economic losses (Sitjà-Bobadilla and Palenzuela, 2012). *E. leei* presents a wide geographical distribution and host range (Padrós et al., 2001; Diamant et al., 2006; Rigos and Katharios, 2010; Katharios et al., 2011, 2014; Sitjà-Bobadilla and Palenzuela, 2012), although its virulence varies depending on the infected species. The disease shows a severe clinical picture and high mortality in some cases, such as for *Diplodus puntazzo* or *Takifugu rubripes* (Yanagida et al., 2006; Álvarez-Pellitero et al., 2008), but it can also develop as a chronic condition, with progressive emaciation and low mortality of diseased fish, as observed in gilthead seabream *Sparus aurata* (Fleurance et al., 2008; Sitjà-Bobadilla et al., 2008).

Similarly, turbot enteromyxosis caused by *E. scophthalmi* is clinically characterized by a cachectic syndrome, being anorexia, weight loss and lethargy the main symptoms (Branson et al., 1999; Sitjà-Bobadilla et al., 2006; Bermúdez et al., 2010; Sitjà-Bobadilla and Palenzuela, 2012). A decrease in hematocrit values, consistent with anemia, has also been reported (Bermúdez, 2003; Sitjà-Bobadilla and Palenzuela, 2012). The fecal–oral route, through the ingestion of the infective stages present in the stools of diseased fish, is thought to be the main route of entry of the myxozoan (Redondo et al., 2004). This way of transmission favors the rapid spread of enteromyxosis among the productive units: infected turbot are considered the primary source of transmission and the high-density culture represents a significant risk factor (Redondo et al., 2002; Quiroga et al., 2006).

The experimental transmission of enteromyxosis by waterborne contamination from the effluent of a tank containing infected fish or by cohabitation of infected and test fish are likely the ways that best reproduce the situation of spontaneous infections among cultured turbot (Redondo et al., 2002, 2004; Bermúdez et al., 2006b; Sitjà-Bobadilla et al., 2006; Losada et al., 2012). On the other hand, these are slower and more heterogeneous infection models than experimental *per os* transmission; the parasite is firstly detected by histology from 20 days post-exposure onwards in infections by effluent or cohabitation, while it can be observed as early as 7 days after experimental *per os* inoculation (Redondo et al., 2004; Bermúdez et al., 2006b; Sitjà-Bobadilla et al., 2006; Losada et al., 2014a). The experimental infection by oral route is considered the most effective way for infecting turbot, with more homogeneous prevalence rates and lesions (Redondo et al., 2002, 2004), allowing the selection of a proper number of specimens with analogous lesions for case-control studies involving gene expression and/or immunohistochemical marker analyses (Ronza et al., 2015a). In all the challenges described, no matter the experimental infection method employed, prevalence and mortality rates often reached 100%, reflecting the high susceptibility of turbot to the infection also observed in spontaneous outbreaks (Redondo et al., 2002, 2004; Bermúdez et al., 2006b; Sitjà-Bobadilla et al., 2006; Losada et al., 2014a).

**FIGURE 1:**
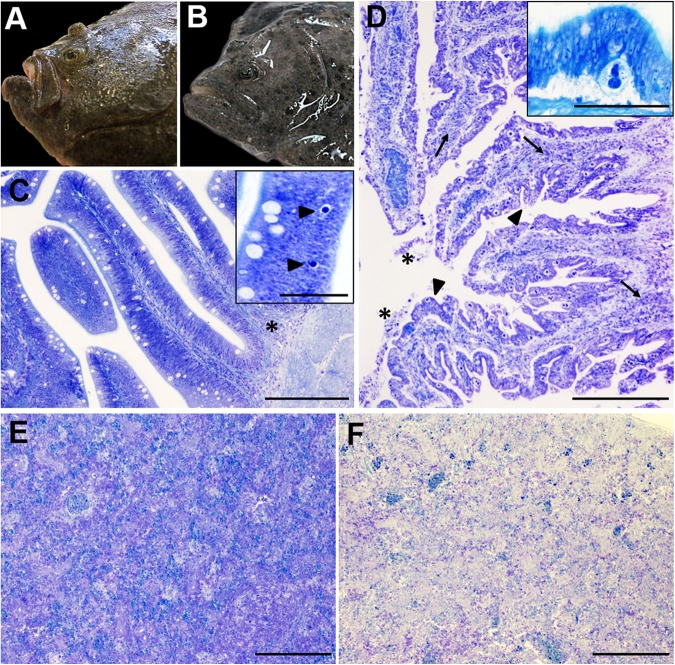
Gross **(A,B)** and microscopic **(C–F)** pathology of turbot enteromyxosis. **(A,B)** Comparative photographs of a healthy turbot **(A)** and a specimen infected by *Enteromyxum scophthalmi* **(B)**. Note the prominence of the head bony ridges in the diseased turbot associated to muscle atrophy, which constitute the characteristic external lesion of enteromyxosis named as “sunken head.” **(C)** Photomicrograph of turbot pyloric caeca at early stages of the parasitization. The intestinal architecture is maintained, subtle alterations such as slight mononuclear infiltrates (asterisk) and the presence of few early developmental stages of *E. scophthalmi* (arrowheads, inset) are observed. **(D)** Photomicrograph of turbot pyloric caeca at advanced stages of the parasitization. There is moderate to severe inflammatory infiltration of the lamina propria-submucosa (arrows) and presence of desquamated cells in the lumen (asterisks). The intestinal epithelium shows a typical “scalloped” shape (arrowheads) and harbors an elevated number of parasitic structures (see the inset, presenting a high magnification of a developmental stage 3 of *E. scophthalmi*). **(E,F)** Comparative photographs of the spleen in a control **(E)** and a severely infected **(F)** turbot, with an evident cell depletion in the latter. **(C–E)** Toluidine blue stain, bars = 200 μm (insets bars = 50 μm).

The disease presents a chronic course, and the progressive emaciation is externally reflected by enophthalmos and conspicuous head bony ridges, due to muscle atrophy (Branson et al., 1999; García, 1999; Bermúdez et al., 2010; Losada et al., 2014a). For this reason, it was initially named as “sunken head” syndrome (Figure 1A,B). Ascites and dilated, congestive or even hemorrhagic alimentary canal, containing a seromucous liquid, are often reported at necropsy (Branson et al., 1999; García, 1999; Bermúdez et al., 2010; Losada et al., 2014a). Pale appearance of other organs and/or splenomegaly was sporadically described, but often there are no significant macroscopic lesions outside the gastrointestinal tract (Branson et al., 1999; García, 1999; Bermúdez et al., 2010).

### Histopathology

The gastrointestinal tract, where the trophozoites of *E. scophthalmi* develop in the lining epithelium, shows the most characteristic microscopic lesions. The disease is typically defined by a picture of catarrhal gastroenteritis, its severity increasing throughout the infection and most of the times leading to the death of the fish. Typically, infection begins in pyloric caeca and anterior intestine, extending up- and backward through the alimentary canal, leading to the colonization of the entire gut, from the esophagus to the anus (Redondo et al., 2002, 2004; Bermúdez et al., 2010; Losada et al., 2012). Bermúdez et al. (2010) performed a comprehensive histopathological study of enteromyxosis, analyzing naturally and experimentally infected turbot at different stages of the disease. They proposed a histological grading of enteromyxosis based on the lesional pattern and parasitic load observed (Bermúdez et al., 2010).

In slight infection (Figure 1C), most of the intestinal folds do not show significant alterations or histologically visible parasites. Early developmental stages of *E. scophthalmi* are sporadically observed at the base of the lining epithelium, sometimes associated with slight infiltration of mononuclear immune cells in this site and/or in the lamina propria-submucosa (Bermúdez et al., 2010). The histological detection of early parasitic stages is difficult, as they are small, rounded, basophilic structures, easily confused with apoptotic cells (Redondo et al., 2004; Bermúdez et al., 2010; Losada et al., 2014a). An increased density of mucous and rodlet cells has also been described at this stage (Bermúdez et al., 2010).

Moderate infection is characterized by a notable increase in parasitic load; different development stages of *E. scophthalmi* can be observed through all the alimentary canal, although they are still more frequent in pyloric caeca and anterior intestine. The gland epithelium of the stomach can also be affected. The inflammatory infiltration is evident, although not always related to a high number of parasites. Infiltrates are mainly composed by intraepithelial lymphocytes and mixed inflammatory cells in the lamina propria-submucosa, where the presence of melanomacrophage aggregates has also been occasionally reported (García, 1999; Sitjà-Bobadilla et al., 2006; Bermúdez et al., 2010; Losada et al., 2014b). At this stage the normal architecture of the gut starts to show pathological evidences, represented by a scalloped shape of the lining epithelium (Bermúdez et al., 2010; Losada et al., 2014a).

The lesions extend to most of the digestive tract in severe infection (Figure 1D), when the presence of the Myxozoa is widespread in all the gut regions. The epithelium is often detached from the basal lamina, showing a variable degree of desquamation, and even a total absence of epithelium can be observed in the most serious cases (Branson et al., 1999; García, 1999; Redondo et al., 2004; Bermúdez et al., 2010; Losada et al., 2014a). The enterocytes show severe alterations, such as necrotic or apoptotic features, vacuolated cytoplasm and fragmented nucleus; the apical brush border and cell-cell junctions are often lost. Groups of apoptotic desquamated cells still associated to parasitic forms are often described (Redondo et al., 2004; Bermúdez et al., 2010; Losada et al., 2014a). Different degrees of inflammatory infiltration were also reported in most gut regions, often severe, and leukocytes with apoptotic features are often detected among those constituting the infiltrates (Sitjà-Bobadilla et al., 2006; Bermúdez et al., 2010; Losada et al., 2014a). The activation of tissue repair processes has also been documented, observing areas of re-epithelialization constituted by squamous or low cubic cells (García, 1999; Bermúdez et al., 2010).

In other organs, the most characteristic lesion is the lymphohematopoietic depletion, observed in spleen and kidney (Figure 1E,F). This lesion is always reported in turbot at advanced enteromyxosis stages (Bermúdez et al., 2006b, 2010; Sitjà-Bobadilla et al., 2006; Losada et al., 2014a). Further, increased presence of apoptotic cells and changes in density and morphology of the melanomacrophage centers have also been documented in the same organs (Bermúdez et al., 2006b, 2010; Sitjà-Bobadilla et al., 2006; Ronza et al., 2013a). *E. scophthalmi* has occasionally been detected in locations other than the gastrointestinal tract, such as skin and gills (other possible routes of entry), blood (possible dissemination route) and lymphohematopoietic organs, sometimes engulfed by macrophages (Redondo et al., 2002, 2004; Sitjà-Bobadilla et al., 2006; Bermúdez et al., 2010; Estensoro et al., 2014). There are also anecdotic descriptions of the parasite presence in bile ducts, pancreas and muscle, in cases of severe infection with an extremely high parasite load (García, 1999; Redondo et al., 2004; Bermúdez et al., 2010). The extraintestinal localization of *E. scophthalmi* is usually not associated with histological alterations.

### Host-Parasite Interaction: Morphopathological Studies

Until recently, host-parasite interaction in turbot enteromyxosis was mainly analyzed through morphological techniques. Light and electron microscopy were used to study the location of the parasite and the lesions associated. *E. scophthalmi* colonizes the digestive tract invading the lining epithelium, where it localizes between the mucosal cells, establishing connections with them through cytoplasmic projections and cell-cell junctions. These structures are thought to be related with mechanisms for attachment, communication and nutrition of the trophozoites (Redondo et al., 2003b; Bermúdez et al., 2010). Intracellular early developmental stages of *E. scophthalmi* have occasionally been described (Palenzuela et al., 2002; Redondo et al., 2003b, 2004). *In vitro* assays using intestinal explants showed the capability of the myxosporean to invade the epithelium both by its apical or basal surface (Redondo et al., 2004).

Lectin- and immune-histochemistry have also been widely employed to deepen into the knowledge of turbot-*E. scophthalmi* interaction. The role of carbohydrate-lectin interactions in the adhesion and penetration of the parasite in turbot epithelium was demonstrated by combining the use of intestinal explants and lectin histochemistry. *N*-acetylgalactosamine, galactose and mannose/glucose residues were identified as the main carbohydrate terminals in the parasite membrane involved in the recognition mechanisms, and the corresponding binding lectins showed an inhibitory effect on its adhesion and penetration (Redondo et al., 2008; Redondo and Álvarez-Pellitero, 2010a,b).

**FIGURE 2:**
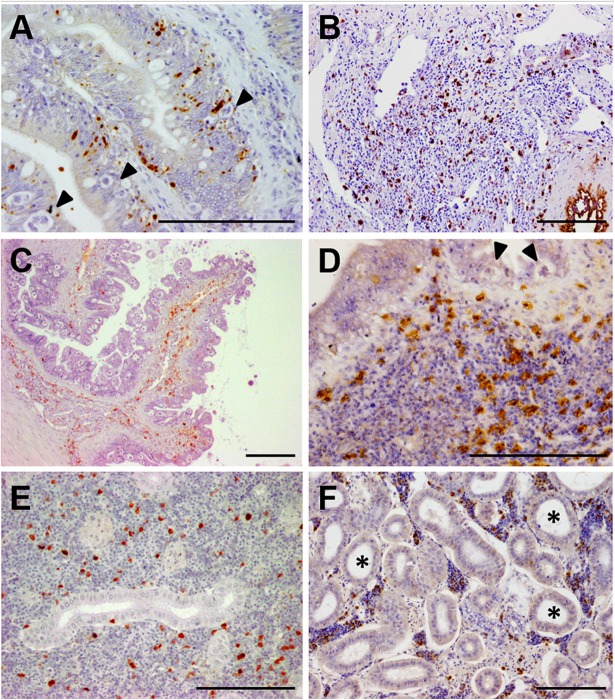
Photomicrographs of illustrative immunohistochemical findings in *E. scophthalmi*-infected turbot. Brown color indicates positive staining, bars = 100 μm. **(A)** Immunostaining of active caspase-3, an indicator of apoptotic cell death, in the intestinal lining epithelium of turbot, where several parasitic structures are visible (arrowheads). **(B)** Inflammatory infiltrate in the lamina propria-submucosa of a diseased turbot, showing an abundant presence of cells immunoreactive to inducible nitric oxide synthase (iNOS). **(C)** Intestinal folds of a heavily parasitized turbot, where numerous IgM-positive (IgM^+^) cells can be observed, mainly in the lamina propria-submucosa, as well as an elevated parasitic load in the epithelium. **(D)** Immunoreactivity to tumor necrosis factor-alpha (TNFα) of several cells constituting the inflammatory infiltrate in the gut of an *E. scophthalmi*-infected turbot. Two parasites (arrowheads) are recognizable in the lining epithelium. **(E)** Turbot kidney showing scattered IgM^+^ in the lymphohematopoietic interstitial tissue of the organ. **(F)** Kidney of turbot with advanced enteromyxosis showing immunostaining to TNFα of some cells of the intertubular parenchyma, which suffered a remarkable cell depletion associated to dilatation of renal tubules (asterisks).

Immunohistochemistry has often been employed as an important complement for the histopathological evaluation of parasitized fish. The presence of the parasite in the intestinal mucosa was associated to the progressive alteration of the lining epithelium, which compromises the proper intestinal barrier function and has been related to disorders in osmoregulation and nutrient absorption. These mechanisms, along with anorexia, would predispose to the wasting syndrome typical of enteromyxosis (Sitjà-Bobadilla and Palenzuela, 2012). In turbot enteromyxosis, the loss of cell-cell junctions in the intestinal mucosa was observed by transmission electron microscopy (Bermúdez et al., 2010) and supported by the immunohistochemical alteration of the expression pattern of several cell junction proteins (Ronza et al., 2013b). Moreover, the observation of increased apoptosis in the gut of diseased fish was confirmed by immunostaining for active caspase-3 (Figure 2A), a crucial mediator of programmed cell death. Apoptotic cells were observed in the lining epithelium and intestinal lumen, often engulfing parasitic structures (Losada et al., 2014a). It has been suggested that this could be beneficial for the host to reduce the parasite load, but *E. scophthalmi* might also take advantage of being eliminated with apoptotic cell remnants to better survive in the water and find a new host (Redondo et al., 2003a; Bermúdez et al., 2010). Bermúdez et al. (2010) also suggested that the apoptosis could be a consequence of the loss of anchorage of the epithelial cells to the extracellular matrix, a mechanism known as anoikis.

Turbot immune response was also primarily investigated by using immunohistochemistry. Immunoreactivity to inducible nitric oxide synthase (iNOS), an important mediator of innate immune response, was notably increased in the gastrointestinal tract of parasitized turbot (Figure 2B), where immune cells, mucous cells and the epithelium itself were labeled. As well, an enhanced number of iNOS-positive cells was found in kidney and spleen of infected fish (Losada et al., 2012). The hypothesis about the relationship of the intestinal lesions with an exacerbated local inflammatory reaction was supported by this study and the investigations on turbot neuroendocrine system (NES). NES plays a key role in the digestive function and alimentary behavior, but it is also involved in the coordination of the immune response through its interactions with the immune system (Palmer and Greenwood-Van Meerveld, 2001). A large set of NES hormones/transmitters was studied in infected and non-infected fish by immunohistochemistry, finding an increased presence of molecules that boost the immune response in the intestine of diseased fish (Bermúdez et al., 2007; Losada et al., 2014b). The implications of the inflammatory reaction in the pathogenesis of diseases associated to catarrhal enteritis are well documented in mammals (Peterson and Artis, 2014; Kamekura et al., 2015; Williams et al., 2015).

The immune adaptive response was also investigated by an immunohistochemical technique targeting turbot IgM (Figure 2C,E). Immunoreactive cells were numerous in spleen and kidney at 20–40 days post-infection (dpi) but decreased in advanced stages of the disease. On the other hand, the number of IgM-positive cells in the gastrointestinal tract increased during the infection until 76 dpi, possibly migrating from the lymphohematopoietic organs (Bermúdez et al., 2006b). Sitjà-Bobadilla et al. (2004) also demonstrated using ELISA that turbot produces specific anti-*E. scophthalmi* antibodies, which in some cases showed a protective effect (Sitjà-Bobadilla et al., 2007); still, most evidences indicated that the humoral immunity is delayed and ineffective in turbot against enteromyxosis (Bermúdez et al., 2006b; Sitjà-Bobadilla et al., 2006).

The leucocytic depletion often reported in advanced enteromyxosis could be an important factor underlying immunodepression and/or failure in the connection between innate and adaptive response (Bermúdez et al., 2006b; Sitjà-Bobadilla et al., 2006). This lesion, observed in spleen and kidney, has been related to the increased apoptosis of immune cells in those organs and in the gastrointestinal tract, where the exacerbated need for cell migration from the lymphohematopoietic organs would contribute to cause the depletion (Bermúdez et al., 2006b, 2010; Sitjà-Bobadilla et al., 2006; Losada et al., 2014a).

Immunohistochemistry was also employed in combination with quantitative PCR (qPCR) to investigate the role of tumor necrosis factor-alpha (TNFα) in the disease (Figure 2D,F), an approach that allowed the simultaneous study of gene expression and protein *in situ* visualization on the same specimens (Ronza et al., 2015a). TNFα is a cytokine involved in a broad spectrum of cellular and organismal responses (Goetz et al., 2004; Hehlgans and Pfeffer, 2005; Parameswaran and Patial, 2010). Its main function as a potent pro-inflammatory mediator was demonstrated in teleost species, and there are many reports on the modulation of *TNFα* under pathological conditions (Montes et al., 2010; Schwenteit et al., 2013; Ma et al., 2014; Pennacchi et al., 2014). An immunohistochemical technique was set up in turbot tissues (Ronza et al., 2015b), which was employed along with *TNFα* expression analysis by qPCR on healthy and *E. scophthalmi*-infected fish (Ronza et al., 2015a). An increased number of immunoreactive cells and up-regulation of *TNFα* was reported in the spleen and kidney of turbot with moderate infection, demonstrating the involvement of the cytokine in triggering the immune response against *E. scophthalmi*. At the intestinal level, a progressive increase of immunoreactive cells was noticed with the progress of the disease, many of which constituted the inflammatory infiltrates in the lamina propria-submucosa. Nevertheless, this increment in labeled cells did not correspond to a significant up-regulation of *TNFα* in the intestine, suggesting the recruitment of leukocytes with a preformed intracellular pool of the cytokine from the lymphohematopoietic organs (Ronza et al., 2015a). TNFα was demonstrated to induce the production of nitric oxide in turbot (Ordás et al., 2007), and, concomitantly, iNOS immunohistochemical expression was increased in the gut of *E. scophthalmi*-infected fish (Losada et al., 2012), indicating a possible relation between the two inflammatory mediators during the disease. The prolonged exposure to inflammation of the gastrointestinal tract could explain the development of the typical intestinal lesions of enteromyxosis, in accordance with what has been reported in different mammalian diseases (Panaro et al., 2007; Chokshi et al., 2008; Bienvenu et al., 2010; Watson and Hughes, 2012; Leppkes et al., 2014).

### Pathogenesis Studies Integrating Morphological and Genomics Approaches

Transcriptional profiling is a powerful tool for the identification of genes and pathways involved in host-pathogen interaction and it is acquiring a pivotal role for understanding the pathogenesis of diseases of fish and shellfish (Qian et al., 2014; Sitjà-Bobadilla et al., 2015; Valenzuela-Miranda et al., 2015; Sudhagar et al., 2018). Particularly, RNA-Seq has emerged as the technology of choice for transcriptomic studies (Wang et al., 2009; Qian et al., 2014) due to its high sensitivity and specificity, and its ability to identify new genes, rare transcripts, alternative splice isoforms, and novel SNPs to be used for association studies (Marioni et al., 2008; Morozova et al., 2009; Nielsen et al., 2011). In turbot enteromyxosis, RNA-Seq analysis was applied to get insights into the early (Ronza et al., 2016) and late (Robledo et al., 2014) stages of the disease. The two studies employed a similar approach: after experimental infection, tissue samples at the same time point (24 dpi for early infection and 42 dpi for late infection) were taken for the application of histological and transcriptomic techniques; after a histopathological evaluation and classification of the specimens, infected fish showing similar lesions and their respective controls were chosen for RNA-Seq analysis. In both cases the transcriptomic study was carried out on pyloric caeca, the intestinal region where the infection usually starts (Redondo et al., 2004), and spleen and kidney, the two major lymphohematopoietic organs (Table 1). A meticulous histopathological evaluation of the fish was a valuable tool for obtaining accurate and useful transcriptomic data, while reducing the biological noise and accordingly the number of samples to be tested, and all in all, it was essential for a better understanding of host-parasite interaction and pathogenesis studies.

**Table 1:** RNA-Seq statistics for the three studied organs in *Enteromyxum scophthalmi*-infected and their respective controls at 24 and 42 days post-infection (dpi).

	24 dpi (Ronza et al., 2016)			42 dpi (Robledo et al., 2014)		
		
	Reads per sample	Trimmed and aligned	DE genes	Reads per sample	Trimmed and aligned	DE genes
Head kidney	14.54M	79.44%	287	17.43M	82.16%	1316
Spleen	13.96M	78.85%	211	16.39M	80.06%	1377
Pyloric caeca	14.02M	85.47%	187	17.09M	58.39%	3022


#### Advanced Infection

**FIGURE 3:**
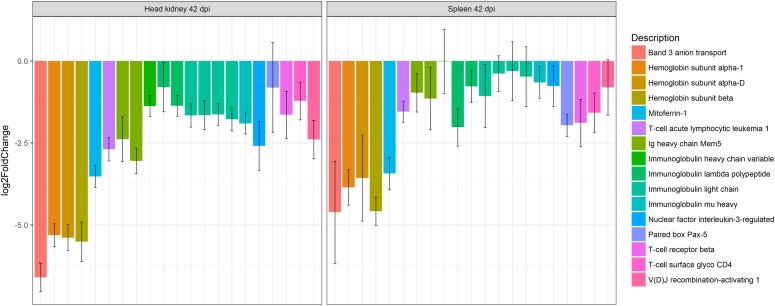
Barplot showing the log2 fold change differences between control and *E. scophthalmi*-infected turbot in head kidney and spleen at 42 days post-infection for genes involved in erythropoiesis and adaptive immune response. The expression profile reflects the lymphohematopoietic depletion observed in turbot with advanced enteromyxosis. Error bars indicate ± SE.

RNA sequencing analysis of severely infected turbot indicated that an exacerbated local inflammatory response is implied in the development of the intestinal lesions. Several proinflammatory genes were found up-regulated, while various genes related to antioxidant defense were down-regulated (Robledo et al., 2014). Oxidative stress linked to prolonged inflammation plays a major role in the pathogenesis of gastrointestinal diseases (Bhattacharyya et al., 2014). The transcriptomic profiling of pyloric caeca also showed the up-regulation of different proapoptotic genes, including caspase-3 (Robledo et al., 2014), in accordance with the immunohistochemical results reported for this protein by Losada et al. (2014a). These authors also described immunoreactive apoptotic cells among those constituting the inflammatory infiltrates at intestinal level, suggesting local immune cell death as a possible reason for an increased cell demand from the lymphohematopoietic organs responsible for the observed leucocytic depletion (Losada et al., 2014a). The involvement of a systemic response during enteromyxosis has been widely demonstrated, by hematological and serological studies (Sitjà-Bobadilla et al., 2006), as well as by immunohistochemistry for IgM (Bermúdez et al., 2006b), iNOS (Losada et al., 2012), and TNFα (Ronza et al., 2015a). Nevertheless, it was highlighted by several authors that at advanced stages of the disease the immune response appears depressed, showing lymphocytopenia (Sitjà-Bobadilla et al., 2006) and decreased numbers of IgM and TNFα immunoreactive cells (Bermúdez et al., 2006b; Ronza et al., 2015a), which has been related to the cell depletion suffered by kidney and spleen. RNA-Seq analysis revealed that not only numerous genes related to adaptive immunity [e.g., immunoglobulin light chain, immunoglobulin mu heavy; V (D) J recombination-activating 1; T-cell surface glycoprotein CD4, T-cell receptor beta chain] were down-regulated in spleen and kidney (Figure 3), but also many genes involved in the coordination between innate and adaptive immunity, such as those related with antigen presenting cells, Th17 lymphocytes and interferons (Robledo et al., 2014). Those results support the hypothesis of a failure in the development of a coordinated immune response of turbot against the disease, where the leucocytic depletion of the lymphohematopoietic organs possibly plays an important role.

Regarding the causes underlying the pathogenesis of the cell depletion, in addition to the exacerbated leukocyte recruitment to the intestine (Bermúdez et al., 2006b; Losada et al., 2014a), the cell death affecting spleen and kidney during the infection has been proposed (Bermúdez et al., 2006b; Sitjà-Bobadilla et al., 2006), but the transcriptomic profiling did not confirm these hypotheses, and a balance between cell death/survival signals was essentially detected in both organs (Robledo et al., 2014).

On the other hand, the transcriptomic analysis highlighted that the spleen and kidney showed down-regulation of genes related with erythropoiesis (Robledo et al., 2014; Figure 3), a finding in accordance with previous observation of anemic status of fish suffering severe enteromyxosis (Bermúdez, 2003; Sitjà-Bobadilla and Palenzuela, 2012). The concurrent down-regulation of ferritin, an important iron-storage protein, and up-regulation of hepcidin, a major regulator of iron metabolism involved in iron sequestration during infections, pointed toward a reduced availability of this element during infection (Robledo et al., 2014). Hepcidin acts as an acute-phase protein during infection, reducing iron absorption in the intestine and iron sequestration in macrophages, thus limiting its availability for hemoglobin synthesis in maturing erythrocytes. This mechanism is considered responsible for the so called “anemia of chronic disease” (Ganz, 2002, 2011). Additionally, it is well known that turbot shows anorexia and severe intestinal lesions at this stage of the disease (Bermúdez et al., 2010; Sitjà-Bobadilla and Palenzuela, 2012), which might affect intestinal iron absorption. Furthermore, in mammals it has been shown that the signaling pathways activated in chronic inflammation affect hematopoiesis (Schuettpelz and Link, 2013), and TNFα, overrepresented in diseased turbot (Ronza et al., 2015a), is thought to have a main role as positive or negative regulator of lymphohematopoiesis (Schuettpelz and Link, 2013; Waters et al., 2013). These mechanisms should deserve further attention as possibly implicated in the cell depletion of lymphohematopoietic organs during enteromyxosis. Interestingly, *TNFα* was not up-regulated in spleen nor in kidney of gilthead sea bream parasitized by *E. leei* (Sitjà-Bobadilla et al., 2008; Pérez-Cordón et al., 2014), and cell depletion of these organs during the disease is not described in this species. Nonetheless, *E. leei*-infected gilthead sea bream also showed an intense local response in the gastrointestinal tract associated to up-regulation of *TNFα* (Davey et al., 2011; Pérez-Cordón et al., 2014). The different entity of the intestinal lesions between these two species, and consequently of the disease course, might be explained by an efficient activation of anti-inflammatory mechanisms in sea bream (Sitjà-Bobadilla et al., 2008; Davey et al., 2011; Pérez-Cordón et al., 2014), which appeared either to fail or to be absent in turbot based on RNA-Seq data (Robledo et al., 2014).

The observation of clinical signs characteristic of a cachectic syndrome (anorexia, weight loss and muscle atrophy) is a common feature of *Enteromyxum*-infected fish (Sitjà-Bobadilla and Palenzuela, 2012). The interaction between the immune response and the NES, through the action of intestinal peptides, has been investigated as an underlying pathogenic mechanism (Bermúdez et al., 2007; Estensoro et al., 2009, 2011; Losada et al., 2014b). Proinflammatory molecules have demonstrated effects as mediators of cachexia in mammals, modulating the production of hormones and neuromodulators, which finally alter the metabolism and feeding behavior causing anorexia, weight loss and tissue wasting (Morley et al., 2006; Tizard, 2008; Grossberg et al., 2010; Freeman, 2012). In severely infected turbot, the transcriptomic profile of the intestine showed, in addition to inflammation, a modulated expression of genes encoding orexigenic and anorexigenic neuropeptides, indicative of the development of anorexia (Robledo et al., 2014). Further, the tissue wasting associated to cachectic syndromes was reflected by a wide down-regulation of genes related to structural proteins in kidney, spleen and pyloric caeca. The anorexic status of diseased fish along with an impaired nutrient absorption caused by the intestinal lesions have been related to reduced synthesis of structural proteins (Robledo et al., 2014), as observed in other species (Wykes et al., 1996; Lenaerts et al., 2006).

#### Early Infection

When RNA-Seq was applied on the same three organs to study the early phase of the disease (Ronza et al., 2016), as expected, a remarkable difference in the number of differentially expressed genes (DEGs) was found in comparison with the advanced stage. In the latter case 1,316 (kidney), 1,377 (spleen), and 3,022 (pyloric caeca) DEGs were found (Robledo et al., 2014), while the numbers were 287, 211, and 187, respectively, in slightly infected turbot (Ronza et al., 2016). These fish were selected for showing incipient signs of the disease, with very subtle histological lesions, such as a slight mononuclear infiltration, and the presence of early developmental stages of *E. scophthalmi* assessed by immunohistochemistry (Ronza et al., 2016).

Differentially expressed genes in common between the two analyzed time points were detected, such as the up-regulation of *CD209* in pyloric caeca (Robledo et al., 2014; Ronza et al., 2016). The protein encoded is a C-type lectin receptor, a surface antigen characteristic of dendritic cells acting in pathogen recognition (Osorio and Reis e Sousa, 2011; Shao et al., 2015). RNA-Seq analysis of severely diseased fish also revealed DEGs related to lectin complement pathway and other C-type lectins (Robledo et al., 2014), supporting the previously hypothesized role of lectins in recognizing *E. scophthalmi* (Redondo et al., 2008; Redondo and Álvarez-Pellitero, 2010b).

**FIGURE 4:**
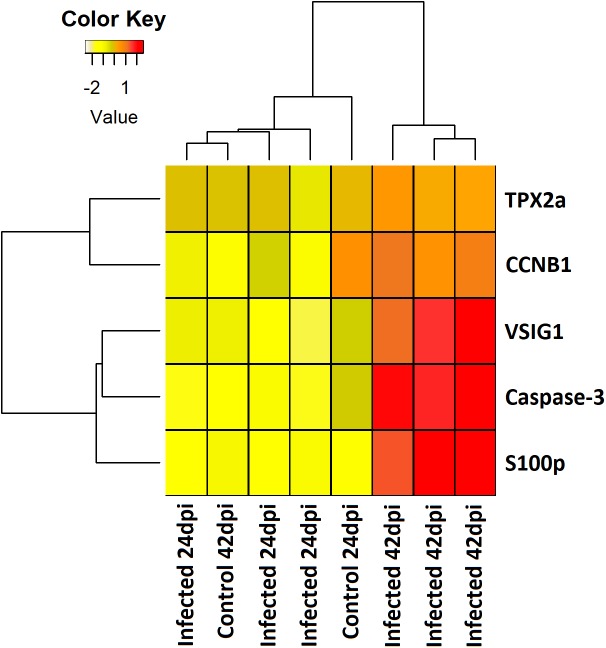
Heatmap showing the expression level (mean centered regularized log transformed normalized counts) of five differentially expressed genes involved in apoptosis and cell proliferation in pyloric caeca of control and *E. scophthalmi*-infected fish at 24 and 42 days post-infection. *TPX2a*, targeting protein for Xklp2-A; *CCNB1*, G2/mitotic-specific cyclin-B1; *VSIG1*, V-set and immunoglobulin domain-containing protein 1; *S100p*, S100 calcium binding protein P.

Mechanisms underlying the adhesion and penetration in the intestinal epithelium by *E. scophthalmi*, a key factor for the course of the infection, were suggested by the modulation of a set of genes at the early stages. These included the cytoskeletal remodeling of host enterocytes through genes acting in the c-Jun N-terminal protein kinases (JNK) pathway, and the alteration of the physiological renewal of the intestinal lining epithelium by inhibiting apoptosis and cell proliferation (Ronza et al., 2016; Figure 4). Other pathogens show similar strategies, such as the intestinal parasite *Cryptosporidium parvum*, which induces a biphasic modulation of apoptosis consisting of early inhibition and late promotion of cell death (Liu et al., 2009). In enteromyxosis, the inhibition of apoptosis and cell proliferation at early stages would favor the epithelial invasion and proliferation of the parasite (Ronza et al., 2016), while the apoptotic cell death in advanced infection (Losada et al., 2014a; Robledo et al., 2014) could facilitate the dispersion of *E*. *scophthalmi* and even its survival in the aquatic milieu surrounded by cell remnants (Redondo et al., 2003a; Bermúdez et al., 2010).

Host-pathogen interactions are also characterized by strategies of the parasite to evade the host immune response. Different parasites, including other myxozoans affecting fish (Lom and Dykova, 2006; Sitjà-Bobadilla et al., 2015), benefit from an intracellular localization. This is still a controversial aspect in the case of *E. scophthalmi*, although there are some reports about early development stages (unicellular) inside host cells (Palenzuela et al., 2002; Redondo et al., 2002, 2004). The transcriptomic analysis of the incipient phase of the infection pointed toward a possible intracellular localization of the parasite, given the evidence of activation of the RIG-I-like receptors (RLRs) pathway found in pyloric caeca (Ronza et al., 2016). This pathway triggers the innate immune response against intracellular pathogens mediated by interferons, inducing the expression of interferon-stimulated genes (ISGs) (Dixit and Kagan, 2013; Nie et al., 2015). A transcriptomic profile consistent with RLRs pathway activation was also observed in spleen and kidney, along with evidences of T cell activation, also related to an interferon-mediated response against intracellular antigens (Ronza et al., 2016). These results suggest that at least some parasite stages enter the blood stream and reach distant organs as kidney and spleen, in accordance with previous observations (Redondo et al., 2003b, 2004).

**FIGURE 5:**
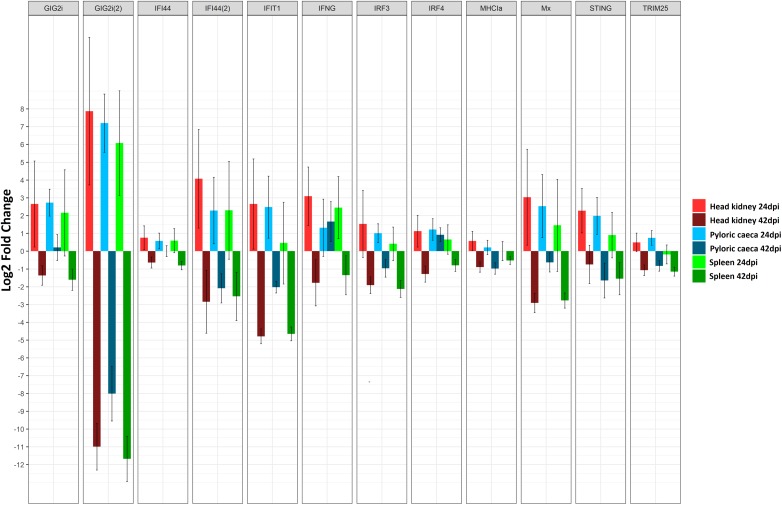
Barplot showing the log2 fold change differences between control and *E. scophthalmi*-infected turbot for three tissues (head kidney, spleen, and pyloric caeca) at two different time points (24 and 42 days post-infection) for a set of differentially expressed genes involved in interferon signaling. Error bars indicate ± SE. *GIG2i*, grass carp reovirus (GCRV)-induced gene 2i; *IFI44*, interferon induced protein 44; *IFIT1*, interferon-induced protein with tetratricopeptide repeats 1; *IFNG*, interferon gamma; *IRF3*, interferon regulatory factor 3; *IRF4*, interferon regulatory factor 4; *MHCIa*, major histocompatibility complex class Ia antigen; *Mx*, interferon inducible Mx gene; *STING*, stimulator of interferon genes; *TRIM25*, E3 ubiquitin/ISG15 ligase TRIM25.

Interestingly, the opposite expression pattern was found at the advanced stage of enteromyxosis, with down-regulation of interferon-related pathways in the three studied organs (Robledo et al., 2014; Figure 5). This result suggests that the immune response to *E. scophthalmi* is elicited differently during the two stages of infection, perhaps depending on a change in the localization of the parasite during the infection. On the other hand, it might be indicative of the inability to develop an effective immune response, possibly parasite-induced as an immune evasion mechanism, often described in mammalian viral diseases (Gale and Sen, 2009; Song et al., 2013; Taylor and Mossman, 2013). The role of interferon-mediated immune response in parasitic disease has also been highlighted in several investigations in both fish and mammals (Álvarez-Pellitero, 2008; McCall and Sauerwein, 2010; Beiting, 2014; Sitjà-Bobadilla et al., 2015). The inhibition of this response has been related to susceptibility to amoebic gill disease in salmon (Young et al., 2008), while the up-regulation of ISGs has been related with resistance to *E. leei* in gilthead sea bream (Davey et al., 2011). In the case of turbot, a correlation might exist between the wide down-regulation of interferon-mediated response during the advanced stage of enteromyxosis and the high susceptibility of this species to the disease.

Other changes in gene expression indicative of inhibition of the immune response were found at early stages of the infection (Ronza et al., 2016). Although the IFN-mediated response was up-regulated in the three organs, *SOCS1*, a recognized inhibitor of interferon-mediated pathways (Skjesol et al., 2014), was up-regulated in kidney. Similarly, *CD2*, encoding a surface antigen of T and NK cells, was down-regulated in kidney and pyloric caeca (Ronza et al., 2016). The down-regulation of *CD2* was found associated to parasitization by *Leishmania donovani* in humans, being related to disorders in T cell function (Bimal et al., 2008). Hence, although there are strong evidences of activation of certain mechanisms for parasite elimination in early enteromyxosis (such as IFN-mediated response), these might be counteracted thus failing to stop disease progression (Ronza et al., 2016). In the same way, RNA-Seq analysis also suggested that the acute-phase response is targeted by immune evasion strategies of *E. scophthalmi* (Ronza et al., 2016). Acute-phase response is an evolutionary conserved immune mechanism consisting in the adaptive regulation of the synthesis and blood circulation of different proteins in response to most forms of inflammation, infection and tissue injury (Bayne and Gerwick, 2001). This response was found activated during infection in several teleost species, also in case of parasitization (Khoo et al., 2012; Kovacevic et al., 2015). At the early stage of enteromyxosis, several genes encoding for acute-phase proteins (APPs) showed a decreased expression, such as complement components, antiproteases and proteins acting in the iron metabolism. The complement and coagulation cascades pathway was significantly down-regulated in the spleen (Ronza et al., 2016). Complement system and iron metabolism are known to be possible targets of pathogen evasion strategies (Zipfel et al., 2007; Ben-Othman et al., 2014; Leon-Sicairos et al., 2015), and the inhibition of antiproteases may contribute to the virulence of the parasite (Armstrong, 2006; Gomez et al., 2014; Sitjà-Bobadilla et al., 2015). These findings point toward the interference of *E. scophthalmi* with the innate immune response of turbot, which would favor the proliferation and dissemination of the parasitic forms in the gastrointestinal tract. The existence of mechanisms of immune silencing is in accordance with the long pre-patent period showed by enteromyxosis. In the study by Ronza et al. (2016), the authors reported that the expression profile of one of the infected fish clustered with the control group for the three studied organs. This fish, although histologically evaluated as early infected considering the absence of lesions and low parasitic load, actually showed more mature and spreading stages of the parasite than the other two infected fish at that stage, suggesting a slightly more advanced infection. This might indicate that the host response is quenched after the early host-parasite interaction (Ronza et al., 2016).

## Conclusion

**FIGURE 6:**
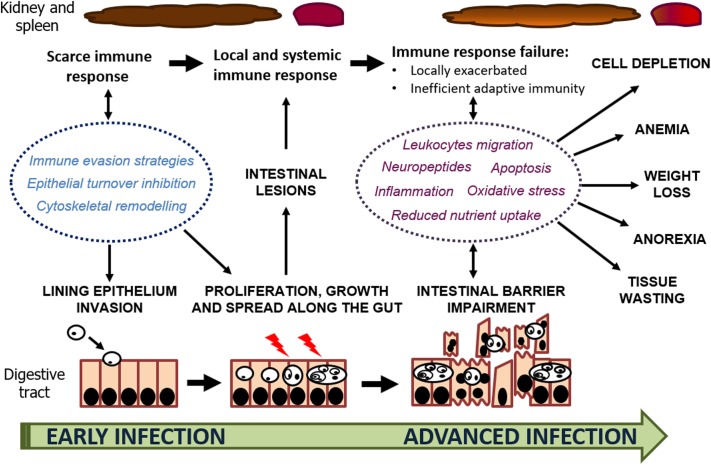
Schematic representation of host-parasite interaction through the progression of enteromyxosis in turbot. The factors involved in the infection mechanisms and development of the lesions and clinical signs are highlighted.

The integrated evaluation of histopathological and transcriptomic information to investigate the pathogenesis of enteromyxosis in turbot provided a comprehensive interpretation of parasite-host interaction (Figure 6) that will aid for developing control measures against this threatening disease. *E. scophthalmi* might benefit from immune evasion strategies to circumvent the turbot immune response at early stages of the infection and reach its target, the gastrointestinal tract. At this level, the remodeling of the host cell cytoskeleton and inhibition of epithelial renewal appear to facilitate the invasion and colonization of the myxozoan parasite. Parasite proliferation might be favored by the silencing of the host immune response, until the parasitic load is increased and the intestinal lesions become serious. The consequent triggering of the inflammatory response would be exacerbated and dysfunctional, failing to coordinate innate and adaptive responses, and lacking an efficient activation of protective anti-inflammatory mechanisms. In this way, turbot immune response would not be able to stop the infection, and instead would contribute to the development of the characteristic clinical signs and lesions associated to the disease.

## Future Perspectives

Myxozoan parasitization in fish is a threat for aquaculture production and there is a need for a more comprehensive understanding of host-parasite interaction, including the entry routes, recognition mechanisms, host immune response and immune evasion strategies (Gomez et al., 2014; Sitjà-Bobadilla et al., 2015). A better characterization and knowledge of the life cycle of these parasites is essential for understanding its epidemiology. In this sense, the inability to culture myxozoans *in vitro* is a major hindrance to get access to a pure source of parasites for experimentation and vaccination, and its proper genetic characterization. Although several methods have been explored to culture *E. scophthalmi in vitro*, including the use of a turbot cell line, none has been successful (Redondo et al., 2003a). The *E. leei* transcriptome has been recently characterized using the intestine of heavily infected fish as source of the parasite (trophozoites) (Shpirer et al., 2014; Chang et al., 2015), and a similar strategy has been recently employed in *E. scophthalmi* with promising results (Palenzuela et al., unpublished data).

Regarding host-parasite interactions in enteromyxosis, the early phase of the infection, which results in epithelial invasion and colonization, is incompletely understood (Sitjà-Bobadilla and Palenzuela, 2012). The application of transcriptomic analysis in turbot enteromyxosis has provided novel and intriguing information on this issue. The identification of the molecular factors and the characterization of the pathways behind disease pathogenesis, clarifying their roles in resistance/susceptibility to the disease, will contribute to the development of more precise predictive tools, control and therapeutic strategies, and in turn, new relevant information for breeding programs.

Given the long pre-patent phase of this disease, early detection tools are a major goal for enteromyxosis control. Therefore, delivering suitable biomarkers for early diagnosis would have a direct impact on disease management strategies. Further, early detection of the parasite would also enable early treatment of the parasitosis, and, in this regard, understanding the early phase of the infection could provide molecular targets for drug development to disrupt parasite invasion. In this sense, the determination of IFNs blood levels or different APPs has been widely used as a diagnostic and prognostic marker for several diseases (Bauer et al., 2006; Schrödl et al., 2016). This is an area that needs to be explored in fish pathology, in combination with high-throughput blood gene expression profiling, which is being successfully applied in human and veterinary medicine to identify markers of health status and infectious and non-infectious diseases (Chaussabel, 2015). Additionally, microRNAs (miRNAs) are emerging as biomarkers for different diseases with a promising diagnostic potential (Wang et al., 2016). A first description of the turbot miRNAs repertoire has been recently published (Robledo et al., 2017b), and new advances on this issue are expected, including the possible applications to disease management.

The integrated analysis of gene expression profiles with the histological lesions associated to disease progression, and the characterization of relevant gene products in the tissue context by other methods (immunological techniques, proteomic analyses) is a promising approach for a comprehensive evaluation of the consistency and significance of the transcriptomic results. Key genes related to response to enteromyxosis identified by RNA-Seq can represent useful intermediate phenotypic markers for direct and more accurate estimations of resistance, which could speed up selective breeding programs. In that sense, despite the hints of potential genetic variation for resistance to *E. scophthalmi*, future quantitative genetics and animal breeding studies are critical to assess this aspect and its correlation with other productive traits in turbot, and, consequently, its potential for selection. Understanding the mechanisms of host-parasite interaction will contribute to selection endeavors through the identification of genes underlying possible QTL and the prioritization of functional markers for genomic selection, potentially leading to an increase of the efficiency and accuracy of selection.

Markers related to the immune system should clearly receive special attention, as they reflect the immunological status of the animal and might indicate the specific traits related to disease resistance/susceptibility. There are some examples of immune indicators that have been used in selective breeding programs for different fish diseases (Das and Sahoo, 2014). Signaling pathways involving IFN-related genes seem to be relevant in turbot early response to enteromyxosis, and the complement system and APP were suggested targets of parasite immune system evasion strategies. Since the liver is the main producer of complement components and APP, the investigation of the hepatic gene expression profiles would help to clarify its role. This strategy can be combined with the search of single nucleotide polymorphisms (SNPs) associated with disease resistance, either on identified candidate genes or detected through genome-wide association analysis (GWAS) using high throughput genotyping approaches (genotyping-by-sequencing or SNP arrays). The investigation of SNP variants has been recently applied in turbot in combination with RNA-Seq to another main target trait for aquaculture production, growth rate (Robledo et al., 2017c).

Finally, all functional and genetic information gained on the progress of enteromyxosis in turbot can feed genome editing approaches in the future. Key genes or genome regions can be modified to (a) confirm their involvement on the parasitosis and (b) develop more resistant animals. While genome editing is obviously a powerful tool and will be key to validate and exploit functional results in the future, this technique has not been tested in turbot and will require an important research effort (and societal changes) before its application in aquaculture.

Nowadays, research in fish pathology benefits from novel and powerful genomic technologies that can be combined with morphopathological approaches to advance in the understanding of diseases’ pathogenic mechanisms. This multidisciplinary approach will be essential for deciphering the physiopathological relevance of the observations and, together with the development of complementary genetic studies, will allow us to acquire new powerful tools for controlling diseases that compromise aquaculture production.

## Author Contributions

PM and MQ conceived the review and participated in the reviewing and critical analysis of the manuscript. PR wrote the original draft and generated part of the figures. DR, BP, RB, and AL participated in writing, preparation of artwork, reviewing and critical analysis of the manuscript.

## Conflict of Interest Statement

The authors declare that the research was conducted in the absence of any commercial or financial relationships that could be construed as a potential conflict of interest.
