# Corrigendum: Integrating Genomic and Morphological Approaches in Fish Pathology Research: The Case of Turbot (*Scophthalmus Maximus*) Enteromyxosis

**DOI:** 10.3389/fgene.2019.00225

**Published:** 2019-03-20

**Authors:** Paolo Ronza, Diego Robledo, Roberto Bermúdez, Ana Paula Losada, Belén G. Pardo, Paulino Martínez, María Isabel Quiroga

**Affiliations:** ^1^Departamento de Anatomía, Producción Animal y Ciencias Clínicas Veterinarias, Universidade de Santiago de Compostela, Lugo, Spain; ^2^Royal (Dick) School of Veterinary Studies, The Roslin Institute, The University of Edinburgh, Midlothian, United Kingdom; ^3^Departamento de Zoología, Genética y Antropología Física, Universidade de Santiago de Compostela, Lugo, Spain

**Keywords:** *Scophthalmus maximus*, Myxozoa, pathogenesis, histopathology, transcriptomics

In the original article, we neglected to include the funder “European Regional Development Fund (ERDF),” which, together with the Spanish Ministry of Economy, Industry and Competitiveness co-funded the projects that supported our work (AGL2015–67039–C3–1–R and AGL2015–67039–C3–3–R).

The authors apologize for this error and state that this does not change the scientific conclusions of the article in any way. The original article has been updated.

